# Possible contribution of pannexin‐1 to ATP release in human upper airway epithelia

**DOI:** 10.1002/phy2.227

**Published:** 2014-02-10

**Authors:** Toyoaki Ohbuchi, Fumiko Takenaga, Nobusuke Hohchi, Tetsuro Wakasugi, Yoichi Ueta, Hideaki Suzuki

**Affiliations:** 1 Department of Otorhinolaryngology‐Head and Neck Surgery, School of Medicine, University of Occupational and Environmental Health, Kitakyushu, Japan; 2 Department of Physiology, School of Medicine, University of Occupational and Environmental Health, Kitakyushu, Japan

**Keywords:** ATP, human upper airway, P2X7 receptor, pannexin‐1 channel

## Abstract

Pannexins are a family of transmembrane nonselective channel proteins that participate in the release of ATP into extracellular space. Previous studies have suggested that pannexin‐1 (Panx1) may constitute a local autocrine/paracrine system via transmitter ATP in association with the purinergic P2X7 receptor. In this study, we investigate the expressions of Panx1 and P2X7 in human nasal mucosa, together with hypotonic stress‐induced ATP release from this tissue. Twenty men and one woman ranging in age from 10 to 82 years with an average age of 44.2 ± 4.4 years participated in the study. Inferior turbinates were collected from patients with chronic hypertrophic rhinitis during endoscopic endonasal surgery. The expressions of Panx1 and P2X7 were examined by fluorescence immunohistochemistry and quantitative reverse transcription‐polymerase chain reaction (qRT‐PCR). We also examined hypotonic stress‐induced ATP release from the turbinate mucosa and the effects of channel blockers in an ex vivo experiment. Substantial expressions of both proteins were observed in human nasal mucosa. The immunoreactivity for Panx1 was stronger than that for P2X7. The presence of the transcripts of Panx1 and P2X7 was also shown by qRT‐PCR. Ten and 100 *μ*mol/L carbenoxolone (a Panx1 channel blocker) significantly inhibited the ATP release from the nasal mucosa, but flufenamic acid (a connexin channel blocker) and gadolinium (a stretch‐activated channel blocker) did not. These results indicate the coexistence of Panx1 and P2X7 in, and Panx1‐dependent ATP release from, the human nasal mucosa, suggesting the possible participation of these molecules in the physiological functions of the upper airway.

## Abstract (precis)

Pannexin‐1 (Panx1) participates in ATP release into the extracellular space. We demonstrated the existence of Panx1 and its ATP‐releasing effect in the human nasal mucosa. Panx1 may be a key molecular component in upper airway physiology.

## Introduction

Pannexins are a family of transmembrane channel proteins in vertebrates homologous to innexins, which are invertebrate gap‐junction proteins (Bruzzone et al. 2003). They comprise three subtypes – pannexin‐1, pannexin‐2, and pannexin‐3 – and have no significant sequence similarity to connexins, the prototypical vertebrate gap‐junction proteins (Yen and Saier 2007). The pannexin membrane channel exists as a homohexamer (pannexin‐1, and probably pannexin‐3) or homooctamer (pannexin‐2) (Boassa et al. 2007; Ambrosi et al. 2010). Although the pannexin genes were originally cloned as gap‐junction‐related proteins, their role as gap‐junction proteins has not been recognized in vivo (Praetorius and Leipziger 2009; Ransford et al. 2009; MacVicar and Thompson 2010; Sosinsky et al. 2011).

The pannexin‐1 (Panx1) channel is the most thoroughly investigated member of the pannexin family and forms a large‐conductance (~500 pS) nonselective channel (Praetorius and Leipziger 2009; MacVicar and Thompson 2010). The activation of the Panx1 channel participates in ATP release into extracellular space (Praetorius and Leipziger 2009; MacVicar and Thompson 2010). It is thought that the Panx1 channel can be activated in either a calcium‐dependent or calcium‐independent mode. In the calcium‐dependent mode, Panx1 channel opening is typically evoked by signal transduction events following the activation of the ionotropic purinergic P2X7 receptor, a representative purinergic receptor directly coupled with the Panx1 channel (Iglesias et al. 2008; Praetorius and Leipziger 2009; MacVicar and Thompson 2010). In the calcium‐independent mode, the Panx1 channel is triggered by mechanical stimulation, such as hypotonic stress‐induced cell swelling and membrane stretching (Bao et al. 2004; Okada et al. 2006; Ransford et al. 2009). These mechanisms suggest that the Panx1 channel may constitute a local autocrine/paracrine system via transmitter ATP for cell–cell signaling (Praetorius and Leipziger 2009; MacVicar and Thompson 2010).

It is known that in the airway, mucociliary clearance (MCC) is modulated by extracellular ATP through the activation of purinergic receptors on the epithelial cell surface (Majima and Sakakura 1995; Zhang and Sanderson 2003; Chen et al. 2006; Zhao et al. 2012). MCC serves to remove inhaled particulate matter along with secreted mucus, and thereby contributes to host defense mechanisms. Mucociliary dysfunction can be a significant clinical problem, and has been reported in various inflammatory airway diseases (Majima and Sakakura 1995). A previous report demonstrated that inhalation of ultrasonically nebulized distilled water induces rapid ionic and/or osmolar changes in the airway fluid, and may lead to the swelling of bronchial epithelial cells in an animal model (Mochizuki et al. 2002). In other reports, the Panx1 channel was shown to be an important contributor to ATP release in hypotonic stress‐stimulated human bronchial airway epithelia (Seminario‐Vidal et al. 2011; Sandilos and Bayliss 2012). These reports led us to hypothesize that the swelling of upper airway epithelial cells via various stimuli induces ATP release through the activated Panx1 channel on the mucosal surface.

The expression and ATP‐releasing effect of the Panx1 channel in the human upper airway epithelia have not yet been investigated. Our previous immunohistochemical and molecular biological studies provided evidence that Panx1 is the predominant pannexin subtype in the rat upper airway (Ohbuchi et al. 2013). In the present study, we investigate the expressions of the Panx1 channel and P2X7 receptor in human nasal mucosa by fluorescence immunohistochemistry and quantitative reverse transcription‐polymerase chain reaction (qRT‐PCR). In addition, we examine whether the Panx1 channel contributes to ATP release from the human nasal mucosa in an ex vivo experiment.

## Material and Methods

### Patients and sample collection

Inferior turbinate samples were collected from 21 patients with chronic hypertrophic rhinitis who underwent inferior turbinectomy via a transnasal endoscopic approach under general anesthesia in our institute. Twenty men and one woman ranging in age from 10 to 82 years with an average age of 44.2 ± 4.4 years participated in the study. Chronic hypertrophic rhinitis was diagnosed by clinical history, rhinoscopic examination, and computed tomography. Informed consent was obtained from all patients. This study was approved by the Ethics Committee of Experimentation of University of Occupational and Environmental Health.

### Fluorescence immunohistochemistry

The nasal mucosa tissue were fixed with 4% paraformaldehyde in 0.1 mol/L phosphate buffer of pH 7.4 (PB) at 4°C overnight. The fixed samples were transferred into 20% sucrose in 0.1 mol/L phosphate‐buffered saline of pH 7.4 (PBS), and incubated at 4°C for 2 nights with 3–4 changes of the solution. The samples were then embedded while frozen in the Tissue‐Tek O.C.T. Compound (Sakura Finetek, Tokyo, JP) and stored at −80°C before sectioning. Seven‐*μ*m‐thick sections were prepared using a cryostat, mounted on saline‐coated glass slides (Superfrost; Matsunami Glass Industries, Osaka, JP), and air‐dried. The sections were hydrated in PBS with 0.3% Triton X‐100 (PBST) for 20 min, and treated with 1.5% normal donkey serum in PBST for 1 h. They were then incubated with rabbit anti‐human Panx1 polyclonal antibody (1:250) (Pinheiro et al. 2013) and goat anti‐human P2X7 receptor polyclonal antibody (1:100) (all from Abcam, Cambridge, U.K.) and diluted in PBST containing 0.5% bovine serum albumin (BSA) at 4°C overnight. After a brief rinse with PBST, the sections were reacted with Alexa Fluor 488‐conjugated donkey anti‐rabbit IgG and Alexa Fluor 568‐conjugated donkey anti‐goat IgG (all from Invitrogen, Molecular Probes, Eugene, OR), then diluted 1:500 in PBST containing 0.5% BSA at room temperature for 2 h. The sections were coverslipped with Prolong Gold antifade reagent containing 4′,6‐diamidino‐2‐phenylindole dihydrochloride (DAPI) (Invitrogen). Images were acquired using a fluorescence microscope ECLIPS TE2000‐U (Nikon, Tokyo, JP) with the confocal laser system BioRad Radiance 2100 (Bio Rad Laboratories Inc., Hercules, CA). The excitation light was a 488‐nm argon laser for Alexa Fluor 488, a 543‐nm helium–neon laser for Alexa Fluor 568, or a 405‐nm blue laser for DAPI. Emitted fluorescence was passed through a 500–530 nm bandpass filter for Alexa Fluor 488, through a 570–700 nm bandpass filter for Alexa Fluor 568, or through a 420–460 nm bandpass filter for DAPI. Captured images were analyzed using the software Bio Rad LaserSharp 2000 (Bio Rad Laboratories Inc., Hercules, CA) and Carl Zeiss LSM Image Browser (Carl Zeiss Co. Ltd, Jena, DE). As a negative control, the primary antibodies were omitted from the process.

### qRT‐PCR

For qRT‐PCR, the specimens were soaked in an RNA stabilization reagent (Qiagen Inc., Valencia, CA) at 4°C overnight. Total RNA was extracted using an RNAqueous‐4PCR Kit (Qiagen Inc.,) according to the manufacturer's instructions. The purity of the RNA was assessed by the ratio of light absorption at 260 to 280 nm (an A_260_/A_280_ ratio in the 1.9–2.1 range was considered acceptable). RNA concentration was determined from A_260_.

Total RNA was reverse transcribed to cDNA with a High‐Capacity RNA‐to‐cDNA Kit (Applied Biosystems Inc., Foster City, CA), which uses random primers. The qRT‐PCR analysis was performed with an Applied Biosystems StepOnePlus real‐time PCR system using the TaqMan Fast Advanced Master Mix (Applied Biosystems) for *Panx1* mRNA, *P2X7* mRNA, and for *glyceraldehyde‐3‐phosphate dehydrogenase* (*GAPDH*) mRNA as a housekeeping gene, according to the manufacturer's specifications. The TaqMan Gene Expression Assays for *Panx1* (assay identification number: Hs00209790_m1), *P2X7* (assay identification number: Hs00175721_m1), and *GAPDH* (assay identification number: Hs02758991_g1) were purchased from Applied Biosystems. One‐hundred ng/*μ*L of cDNA was mixed with TaqMan Fast Advanced Master Mix with AmpErase (uracil *N*‐glycosylase) and the primer/probe set of the TaqMan Gene Expression Assays. The mixture was then subjected to PCR amplification with real‐time detection. The thermal cycler conditions were as follows: holding at 50°C for 2 min followed by 95°C for 20 sec; this was followed by 40 cycles of two‐step PCR with 95°C for 1 sec and 60°C for 20 sec. Each sample was assayed in duplicate.

The measured threshold cycle (C_*T*_) was normalized by subtracting the C_*T*_ for *GAPDH* of each sample from that for *Panx1* and *P2X7*. From the obtained ∆C_*T*_, the ratio of *Panx1* mRNA and *P2X7* mRNA to *GAPDH* mRNA was calculated as follows:
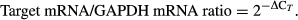


### Measurements of ATP release from nasal mucosa

The lateral surface of the collected inferior turbinate was shaved to prepare a film of the mucosal surface layer immediately after sample collection. The filmy tissue was trimmed with a circular punch (inner diameter = 5 mm), and washed with normal saline. The cutout mucosal segments were incubated in a 12‐well culture plate containing 4 mL isotonic solution (100 mmol/L NaCl with 300 mOsmol L^−1^ adjusted with mannitol) or hypotonic solution (100 mmol/L NaCl with 200 mOsmol L^−1^) in each well at room temperature for 10 min. One‐hundred microliter solution was collected by an ATP water‐testing device, AQUASNAP™ (Hygiena, Camarillo, CA), and the ATP concentration was measured by a luciferin‐luciferase assay using luminometer SystemSURE Plus™ (Hygiena, Camarillo, CA). To assess the contribution of the Panx1 channel to ATP release, the mucosal segment was also incubated in the presence of carbenoxolone (CBX; a Panx1 channel blocker), flufenamic acid (FFA; a connexin channel blocker), or gadolinium (Gd^3+^; a stretch‐activated channel blocker) (all from Sigma, St Louis, MO) (Yang and Sachs 1989; Bruzzone et al. 2005; Ma et al. 2009) at room temperature for 10 min under hypotonic condition. All drugs were dissolved in 100 mmol/L NaCl. The measured ATP concentration was standardized by deducting the ATP concentration before applying the mucosal segment to the solution in each well.

### Statistical analysis

Results were expressed as the mean ± SEM. Statistical comparisons were performed using the Student's *t‐*test, and *P < *0.05 was considered significant.

## Results

### Expressions of Panx1 channel and P2X7 receptor in human nasal mucosa

**Figure 1.**
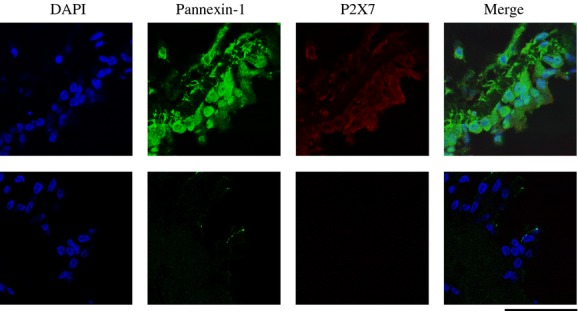
Photomicrographs of fluorescence immunohistochemical staining for Panx1 and P2X7 in human nasal mucosa. The immunoreactivity for both Panx1 and P2X7 is identified in human nasal mucosa. (*n* = 13). The images show positive staining/negative control pairs (upper panels/lower panels). Scale bar = 50 *μ*m.

Figure 1 shows photomicrographs of fluorescence immunohistochemical double staining for Panx1 and P2X7 of the human nasal mucosa (*n* = 13). Substantial expressions of both proteins were identified, including in the epithelial layer. The immunoreactivity for Panx1 was stronger than that for P2X7.

**Figure 2.**
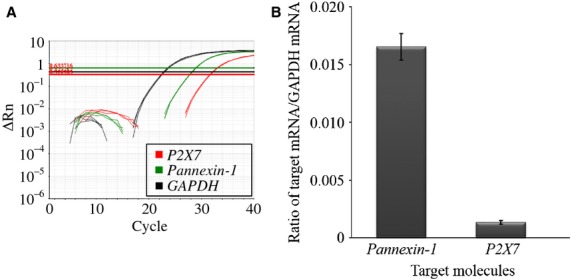
Expression of *Panx1* and *P2X7 *mRNA in human nasal mucosa. (A) The representative trace of amplification plot. (B) Quantity of *Panx1* and *P2X7 *mRNA. The average ratios of *Panx1 *mRNA/*GAPDH *mRNA and *P2X7 *mRNA/*GAPDH *mRNA are 0.0165 ± 0.0011 and 0.0014 ± 0.0002, respectively (*n* = 10).

The qRT‐PCR results are presented in Figure 2. The representative trace of the amplification plot is shown in Figure 2A. This result proves the presence of Panx1 and P2X7 at the transcript level in the human nasal mucosa. The average *Panx1* mRNA/*GAPDH* mRNA and *P2X7* mRNA/*GAPDH* mRNA ratios were 0.0165 ± 0.0011 and 0.0014 ± 0.0002, respectively (*n* =10, Fig. 2B).

### Effects of CBX, FFA, and Gd^3+^ on hypotonic stress‐induced ATP release from human nasal mucosa

**Figure 3.**
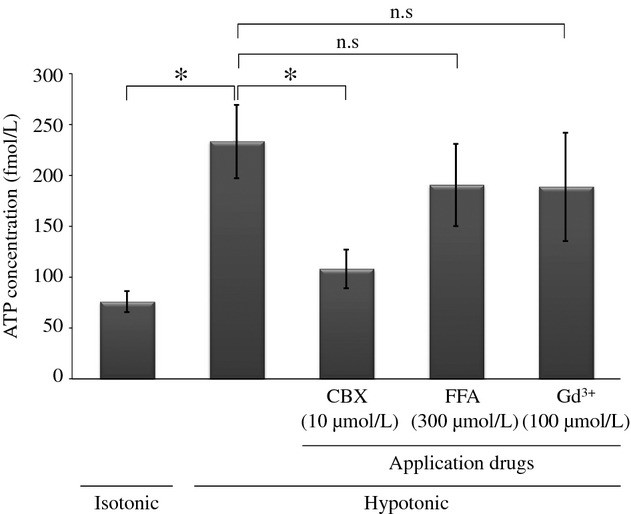
The effects of carbenoxolone (CBX), flufenamic acid (FFA), and gadolinium (Gd^3+^) on ATP release from human nasal epithelia. The mucosal segment cut out from the inferior turbinate was incubated for 10 min. The ATP concentration in the solution was measured by a luciferin‐luciferase assay. The ATP concentration after hypotonic stress was 233 ± 36 fmol/L (*n* = 8), significantly higher than that of isotonic condition (76.0 ± 41 fmol/L, *n* = 3, *P *=**0.0304). In the presence of 10 *μ*mol/L CBX under hypotonic condition, the ATP concentration significantly decreases to 108 ± 19 fmol/L (*n* = 8, *P *=**0.0084). In contrast, the ATP concentration is not inhibited by 300 *μ*mol/L FFA (191 ± 40 fmol/L, *n* = 8, *P *=**0.4444) or 100 *μ*mol/L Gd^3+^ (189 ± 53 fmol/L, *n* = 8, *P *=**0.5004) under hypotonic condition.

The measurements of ATP release from nasal mucosa under various conditions are summarized in Figure 3. The ATP concentration after 10‐min hypotonic stress was 233 ± 36 fmol/L (*n* = 8), significantly higher than that of isotonic condition (76.0 ± 41 fmol/L, *n* = 3, *P *=**0.0304). In the presence of 10 and 100 *μ*mol/L CBX, the ATP concentration significantly decreased to 108 ± 19 (*n* = 8, *P *=**0.0084) and 112 ± 19 fmol/L (*n* = 8, *P *=**0.0105; data not shown), respectively. In contrast, ATP concentration was not inhibited by FFA at a concentration of 300 *μ*mol/L (191 ± 40 fmol/L, *n* = 8, *P *=**0.4444), which is considered sufficient to produce the maximum effect (Bruzzone et al. 2005; Ma et al. 2009). In addition, ATP concentration was not reduced by Gd^3+^ at a concentration of 100 *μ*mol/L (189 ± 53 fmol/L, *n* = 8, *P *=**0.5004). These results suggest that ATP release from the human nasal mucosa under hypotonic condition is mediated, at least in part, by CBX‐sensitive and FFA‐insensitive channels – like the Panx1 channel – and is independent of stretch‐activated channels.

## Discussion

The present study provides the first evidence of the coexistence of Panx1 and P2X7 in the human upper airway. Transcripts for these substances were further quantitated by qRT‐PCR. In addition, CBX inhibited ATP release from human nasal mucosa under hypotonic condition, while FFA and Gd^3+^ did not. These results suggest that the release of ATP from human nasal mucosa may in part be mediated by the Panx1 channel. Obviously, it would not have been ethical to obtain normal inferior turbinates for this study; we therefore collected the turbinates from patients with chronic hypertrophic rhinitis. Although hypertrophied turbinates histologically exhibit irreversible changes such as submucosal fibrosis, the epithelial layer of their lateral side generally shows almost normal histological findings (Berger et al. 2006). Because of this, we deemed the influence of the pathological changes of the hypertrophied turbinate on the function of Panx1 to be negligible.

Gentle nondestructive mechanical stimulation is a common trigger for ATP release from diverse kinds of cells (Praetorius and Leipziger 2009). Typical triggers include gentle rotation of a dish with cultured cells, change in the flow of medium over a sheet of epithelial/endothelial cells, and cell swelling induced by hypotonic stress (Bodin et al. 1991; Grygorczyk and Hanrahan 1997; Mitchell et al. 1998; Koyama et al. 2001; Boudreault and Grygorczyk 2004).

ATP is thought to be released via two distinct pathways; that is, vesicle‐ and channel‐mediated pathways. In the case of the former, it has been observed that ATP release depends partly on an intracellular calcium increase, which is required for vesicular release. As to the latter, the channel‐mediated pathway was discovered by recent work on airway epithelia, which showed that calcium is not needed for hypotonic stress‐induced ATP release. This work also indicated that the Panx1 channel is the most convincing candidate in terms of what mediates this response (Bao et al. 2004; Ransford et al. 2009; Seminario‐Vidal et al. 2011; Sandilos and Bayliss 2012).

Several studies have demonstrated the presence of mechanosensors in the airway mucosa. In the human lower airway epithelia, the Panx1 channel is expressed in the apical membrane, and contributes to ATP release onto the epithelial surface in response to hypotonic stress‐mediated cell swelling (Ransford et al. 2009; Seminario‐Vidal et al. 2011; Sandilos and Bayliss 2012). Our results also indicate that the Panx1 channel is likely to be responsible for ATP release into the extracellular space under hypotonic condition in the human upper airway epithelia. On the other hand, neither Gd^3+^ nor FFA had an effect on ATP release under hypotonic condition, indicating a negligible role of stretch‐activated channels, such as transient receptor potential vanilloid 4, and connexin channels in this reaction in the nasal mucosa (Seminario‐Vidal et al. 2011).

ATP released through the Panx1 channel in response to hypotonic stimulation probably activates the coexisting P2X7 receptor. P2X7 receptor activation is involved in the signal transduction events that lead to the activation of the Panx1 channel; a proline‐rich segment containing a Src homology 3 domain in the carboxyl terminus of the P2X7 receptor regulates Src tyrosine kinase activation, which activates Panx1 (Iglesias et al. 2008). ATP is then released through the Panx1 channel, and the P2X7 receptor is activated again. This positive‐feedback interaction between P2X7 and Panx1 may constitute a local autocrine/paracrine signaling system via ATP‐induced ATP release in the human nasal epithelia.

Src tyrosine kinase activation is dependent on an increase in intracellular calcium brought about by calcium influx though the P2X7 receptor. However, this study demonstrates that the hypotonic stress‐induced pannexin‐dependent ATP release from the nasal mucosa can be observed under a calcium‐free condition, indicating that P2X7 receptor activation plays only a minor role in the initiation of ATP release via the Panx1 channel. However, once the Panx1 channel is activated by other triggers, such as hypotonic stimulus, the reaction is propagated and prolonged via the above positive‐feedback mechanism.

Such a self‐propagating reaction may participate in the generation of intercellular calcium waves; calcium poured into an epithelial cell would spread to contiguous cells through the gap‐junction channels, such as connexin‐43, and elicit another calcium influx reaction in each cell via the same mechanism, leading to the transmission of calcium signaling throughout the airway epithelium (Yeh et al. 2003; Locovei et al. 2006).

What is the role of pannexin‐dependent ATP release in the airway mucosa? Ciliated respiratory epithelial cells increase their ciliary beat frequency upon activation (Sanderson and Dirksen 1986; Sanderson et al. 1990; Cohen 2006), which is a beneficial change for mucus clearance. It has also been reported that mechanical stimulation directly and robustly activates ciliary beat frequency of upper respiratory ciliated cells in vitro, and that this action is mediated by ATP released from the apical surface of the cells (Button et al. 2007; Zhao et al. 2012). These lines of evidence and the present results lead to the hypothesis that ATP released through the Panx1 channel regulates the mechanism of mucus clearance via ciliary beating modulation in the human upper airway.

However, certain issues remain unresolved. First, the specificity of the primary antibodies used in our immunohistochemistry experiment could not be definitively determined. No primary antibody as a negative control is valuable but insufficient. In the current case, siRNA or shRNA knockdown cells for Panx1 or P2X7 would provide the ideal negative controls. Similarly, nonexpressing cells, transfected with the Panx1 or P2X7, would provide the best positive controls. Second, we cannot affirm that hypotonic stress‐mediated effects do not involve as yet unknown molecules other than the Panx1 channel. The blockers used in our protocol do not necessarily exhibit strict pharmacological specificity (Bruzzone et al. 2005; Ma et al. 2009). Third, cell destruction may have affected the ATP measurements. The viability of the mucosal epithelial cells after ATP measurement remains to be examined. Fourth, the physiologic properties of the Panx1 channel in vivo are not fully understood. In culture experiments, ATP concentrations in a medium need to be at a micromolar level for sufficient activation of purinergic receptors (Okada et al. 2006; Ransford et al. 2009), but the ATP concentration in the mucus blanket on the mucosal surface is difficult to determine. In relation to this, we should consider the contributions of other types of purinergic receptors including metabotropic P2Y2 receptor expressed in the airway epithelium to ATP release (Okada et al. 2006; O'Grady et al. 2013).

In conclusion, we evaluated Panx1 and P2X7 expressions in, and ATP release from, the human nasal mucosa by immunohistochemistry, qRT‐PCR, and ex vivo physiological experiment. This is the first report demonstrating the existence of the Panx1 channel and its ATP‐releasing effect in the human upper airway epithelia. The Panx1 channel could be one of the key molecular components in the regulatory mechanism of the upper airway function. Modulation of this molecule may open a novel therapeutic strategy in the management of upper respiratory disorders. Further biochemical, biophysical, and molecular biological evidence is needed to clarify this interesting mechanism in the human nasal mucosa.

## Acknowledgments

We thank Kanji Yahiro and Tomoko Tamura (STEM Biomethod Corporation, Kitakyushu, Japan) for their expertise.

## Conflict of interest

None declared.
